# Interleukin-33/ Suppression of Tumorigenicity 2 in Renal Fibrosis: Emerging Roles in Prognosis and Treatment

**DOI:** 10.3389/fphys.2021.792897

**Published:** 2022-01-03

**Authors:** Xiao-Yang Tan, Hao-Yue Jing, Yue-Rong Ma

**Affiliations:** School of Basic Medicine, Chengdu University of Traditional Chinese Medicine, Chengdu, China

**Keywords:** renal fibrosis, chronic kidney disease, IL-33, ST2, mechanisms

## Abstract

Chronic kidney disease (CKD) is a major public health problem that affects more than 10% of the population worldwide and has a high mortality rate. Therefore, it is necessary to identify novel treatment strategies for CKD. Incidentally, renal fibrosis plays a central role in the progression of CKD to end-stage renal disease (ESRD). The activation of inflammatory pathways leads to the development of renal fibrosis. In fact, interleukin-33 (IL-33), a newly discovered member of the interleukin 1 (IL-1) cytokine family, is a crucial regulator of the inflammatory process. It exerts pro-inflammatory and pro-fibrotic effects *via* the suppression of tumorigenicity 2 (ST2) receptor, which, in turn, activates other inflammatory pathways. Although the role of this pathway in cardiac, pulmonary, and hepatic fibrotic diseases has been extensively studied, its precise role in renal fibrosis has not yet been completely elucidated. Recent studies have shown that a sustained activation of IL-33/ST2 pathway promotes the development of renal fibrosis. However, with prolonged research in this field, it is expected that the IL-33/ST2 pathway will be used as a diagnostic and prognostic tool for renal diseases. In addition, the IL-33/ST2 pathway seems to be a new target for the future treatment of CKD. Here, we review the mechanisms and potential applications of the IL-33/ST2 pathway in renal fibrosis; such that it can help clinicians and researchers to explore effective treatment options and develop novel medicines for CKD patients.

## Introduction

Chronic kidney disease (CKD) is a clinical syndrome with definite changes in kidney structure and function that last for more than 3 months (Ammirati, 2020). Incidentally, CKD is the third leading cause of premature death in patients, following acquired immunodeficiency syndrome and diabetes. In fact, more than 10% of the world’s population is affected by CKD (Lv et al., 2018; Djudjaj and Boor, 2019), thereby making it a major public health problem issue. Interestingly, renal fibrosis occurs in severe cases of CKD, and it is considered as an underlying pathological process of this disease; moreover, renal fibrosis ultimately leads to the progression of CKD to end-stage renal disease (ESRD; Berchtold et al., 2017; Chen et al., 2018a; Martínez-Klimova et al., 2019). Therefore, the underlying mechanisms of renal fibrosis should be extensively studied to ensure a better understanding of CKD development because the inhibition of renal fibrosis may help to decrease the rate of CKD progression.

Fibrosis is a highly coordinated process that occurs as a result of the tissue repair response. Incidentally, the tissue repair response may become dysregulated if the healing response of tissues continues beyond normal wound healing, for instance during chronic inflammation, trauma, infection, metabolic disorders, inflammation, and autoimmunity. This results in the production of cytokines and chemokines. The release of these mediators leads to the local activation of collagen-producing mesenchymal cells, to a transition of various cell types into myofibroblasts as well as to the recruitment of fibroblast precursors (Weiskirchen et al., 2019; Henderson et al., 2020; Zhang and Zhang, 2020). The main pathological characteristics of renal fibrosis are renal interstitial fibroblast hyperplasia and the aberrant and excessive deposition of extracellular matrix (ECM). These changes destroy the normal tubular and interstitial structures of the kidneys (Humphreys, 2018; Zhang et al., 2020). Interestingly, inflammatory responses are central to the progression of renal fibrosis (Zhao et al., 2015; Chen et al., 2019). They involve various cytokine-mediated multi-signaling pathways, including transforming growth factor-beta (TGF-β), tumor necrosis factor-alpha (TNF-α), and leukocyte mediators (Lv et al., 2018; Chen et al., 2018a; Prakoura et al., 2019). Interleukin 33 (IL-33), the gene for which is located on chromosome 9, is a newly discovered member of the IL-1 cytokine family and a pivotal regulator of inflammatory and immune responses (Molofsky et al., 2015; Di Salvo et al., 2018; Chen et al., 2020). It functions in association with the suppression of tumorigenicity 2 (ST2) receptor. In fact, the IL-33/ST2 pathway is involved in causing an imbalance between widespread inflammation and tissue regeneration in several organs, such as the lungs, liver, skin, and gastrointestinal system, ultimately leading to fibrosis (Rankin et al., 2010; Tan et al., 2018; Wu et al., 2018; Imai et al., 2019). Due to the potential role of the IL-33/ST2 pathway in fibrotic diseases, it has received increasing attention. In fact, this review discusses the current progress in research related to the IL-33/ST2 pathway in renal fibrosis to provide new insights into developing treatment options for CKD patients.

## The IL-33/ST2 Pathway

### IL-33

In 1999, IL-33 was first identified in a canine model of arachnoid hemorrhage, which expressed the protein DV27 (Onda et al., 1999). Subsequently, in 2003, endogenous IL-33 and its mRNA were discovered in human tissues, and initially, defined as a nuclear factor from high endothelial venules (NF-HEV; Baekkevold et al., 2003). In 2005, Schmitz et al. (2005) discovered that the hydroxyl-terminal portion of NF-HEV resembles the three-dimensional folded structure of the IL-1 family of cytokines. Moreover, as a cytokine, this factor induces a type 2 immune response by binding to the ST2 receptor. Based on these observations, NF-HEV was classified as a new member of the IL-1 cytokine family, and it was named IL-33 (Schmitz et al., 2005). The IL-33 is a regulator of inflammation, and can induce T-helper 2 (Th2)-mediated innate and adaptive immune responses (Kakkar and Lee, 2008; Kotsiou et al., 2018). Additionally, IL-33 participates in the pathogenesis of renal, neurological, hepatic, pulmonary, and eye diseases. It is a cell cytokine that promotes inflammatory responses and has a characteristic of alarm (Moussion et al., 2008; Li et al., 2019b; Wang et al., 2020). Since the expression of IL-33 has been reported to increase in tissues during inflammation (Kotsiou et al., 2018), IL-33 is a potential mediator of various inflammatory diseases (Dinarello, 2005). Furthermore, IL-33 plays an invaluable role in various biological responses, such as regulation of immune responses, maintenance of tissue homeostasis, and tissue repair and remodeling (Drake and Kita, 2017).

### IL-33 Receptor

The IL-33 receptor complex is a heterodimeric complex consisting of IL-1 receptor accessory protein (IL-1RACP) and ST2 encoded by IL-1receptor type 1(IL1R1; Cayrol and Girard, 2014). In fact, the IL-1RACP functions as a co-receptor for IL-33 signaling (Hong et al., 2019). The ST2, a member of the Toll-like receptor/IL-1 receptor superfamily, has four subtypes, including two variant forms (ST2LV and ST2V), a soluble form (sST2), and a membrane-bound form (ST2L; Bergers et al., 1994; Iwahana et al., 2004; Schmitz et al., 2005; Griesenauer and Paczesny, 2017). The ST2LV is a form of ST2L that is produced by selective splicing (Iwahana et al., 2004). The ST2V is mainly expressed in organs of the digestive system. Its overexpression in cell lines leads to restricted membrane localization (De la Fuente et al., 2015). The most significant subtypes are ST2L and sST2, and they are highly expressed in the kidneys, lungs, placenta, and stomach (Li et al., 2000; Kaur et al., 2020). The ST2L is a functional component of IL-33 signaling, and it promotes inflammation and conducts a Th2-type immune response by activating downstream molecules, such as myeloid differentiation primary response 88 (MyD88) and tumor necrosis factor receptor (TNFR)-associated factor 6 (TRAF6) proteins (Milovanovic et al., 2012; Takeda et al., 2018). The sST2 lacks transmembrane and intracellular domains and can act as a decoy receptor for IL-33 that can help to block IL-33 signaling (Xu et al., 2017; Antunes et al., 2018). It is expressed in mast cells and fibroblasts and its activity is induced by cytokines such as TNF-α.

### IL-33/ST2 Signaling Pathway

**Figure 1:**
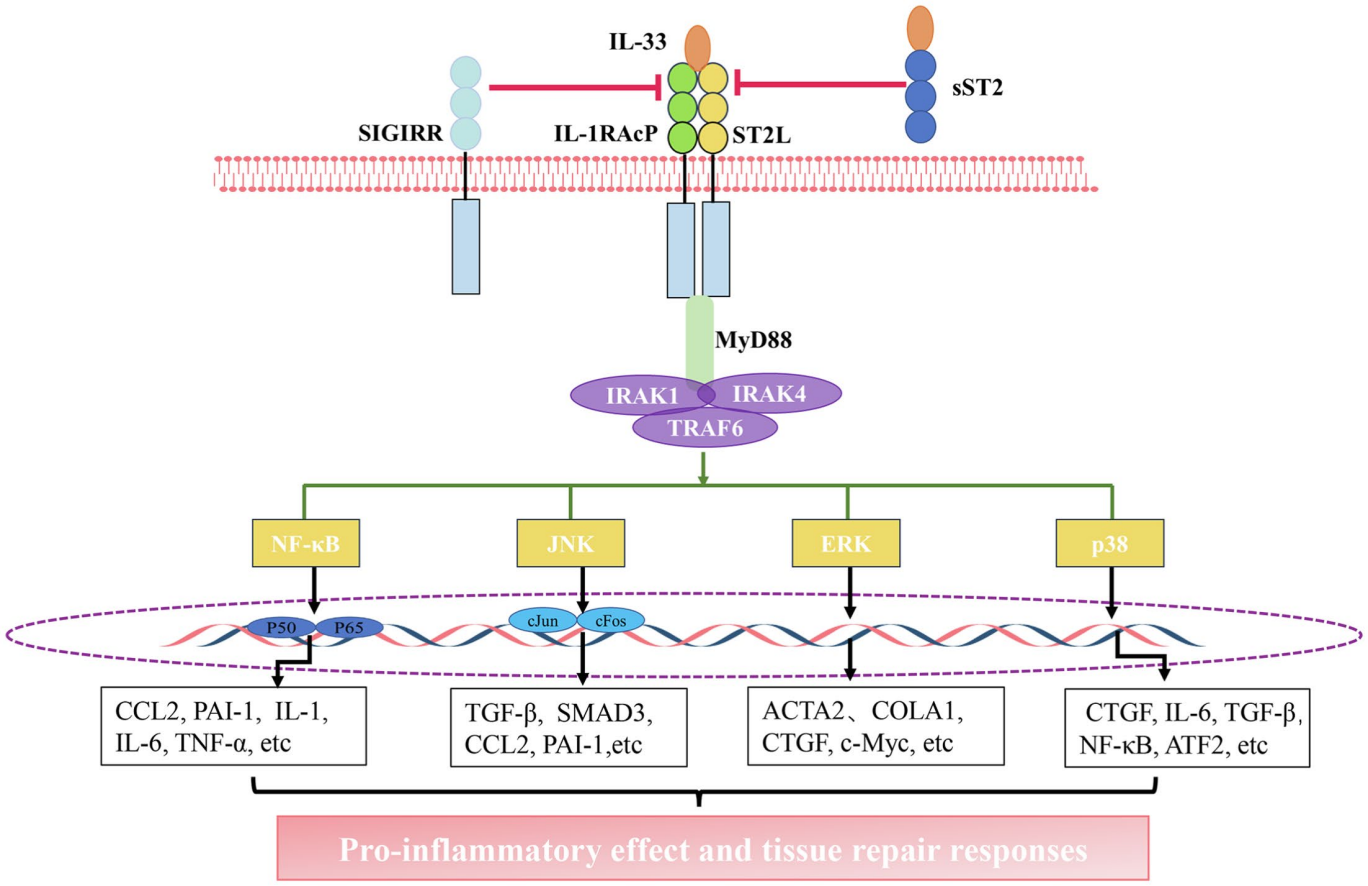
The interleukin-33 (IL-33)/suppression of tumorigenicity 2 (ST2) pathway. Activation of the IL-33/ST2 pathway: IL-33 binds to a receptor complex composed of ST2L and interleukin 1 receptor accessory protein (IL-1RACP) on the cell membrane. The interaction between the C-terminal domain of this receptor complex promotes the recruitment of myeloid differentiation primary response 88 (MyD88), IL-1 receptor-associated kinase 1 (IRAK1), IRAK4, and tumor necrosis factor receptor (TNFR)-associated factor 6 (TRAF6), thus leading to the activation of nuclear factor kappa light chain enhancer of activated B cells (NF-κB), c-Jun N-terminal kinase (JNK), extracellular signal-regulated kinase (ERK), and P38 pathways. The activation of these pathways facilitates the gene expression of chemokines and growth factors. The inhibition of the IL-33/ST2 pathway: soluble ST2 (sST2) acts as a decoy receptor for IL-33 to block the binding of IL-33 to the receptor. single immunoglobulin IL1-related receptor (SIGIRR) can destroy ST2L/IL1RAcP heterodimers.

When cells detect inflammatory signals, or in cases of tissue damage or necrosis occurs, IL-33 is rapidly released from the cell, and it binds to the heterodimeric receptor complex ST2L/IL1-RACP, eventually forming IL-33/ST2L/IL1-RACP complex (Schmitz et al., 2005; Chackerian et al., 2007). Subsequently, MyD88, IL-1 receptor-associated kinase 1 (IRAK1), IRAK4, and TRAF6 are recruited. This leads to the activation of signaling pathways, including nuclear factor kappa light chain enhancer of activated B cells (NF-κB), extracellular signal-regulated kinase (ERK1/2), c-Jun N-terminal kinase (JNK), and p38 mitogen-activated protein kinase (MAPK), ultimately leading to the production of several pro-inflammatory factors (Funakoshi-Tago et al., 2011; Chen et al., 2017; Li et al., 2018; Allegra et al., 2019). However, this pathway can be attenuated through diverse mechanisms. For instance, sST2 can act as a decoy receptor that competes with ST2L to bind with IL-33. Moreover, single immunoglobulin IL1-related receptor (SIGIRR) can also repress this pathway (Gao et al., 2015; Larsen et al., 2018; Shen et al., 2018; Liu et al., 2019d; Figure 1).

## The IL-33/ST2 Pathway and Renal Fibrosis

### Distribution of IL-33 and ST2 in the Kidney

Incidentally, IL-33 is widely expressed in various organs, including the heart, brain, kidneys, liver, spleen, and lungs. At the cellular level, IL-33 is mainly expressed by non-immune cells, including epithelial cells, endothelial cells, and fibroblasts (Schmitz et al., 2005; Martin and Martin, 2016; Chen et al., 2017; Drake and Kita, 2017). In addition, immune cells such as activated macrophages and dendritic cells are also sources of IL-33, but the expression level of IL-33 is low (Li et al., 2014a; Yang et al., 2016). In kidney, immunohistochemical analysis has revealed the presence of IL-33^+^ cells in the renal tubules of the patients with chronic allograft dysfunction (Xu et al., 2018). Similarly, in patients with renal allograft rejection, IL-33 has been detected in the renal tubules and interstitium (Yu et al., 2021). Furthermore, IL-33 is constitutively expressed in endothelial cells of large and small renal vessels (Akcay et al., 2011). Chen et al. (2016) have also reported the presence of a major population of IL-33^+^ cells among α-smooth muscle actin (α-SMA)^+^ interstitial myofibroblasts and a minor population among CD31^+^ peritubular vascular endothelial cells in the kidneys of unilateral ureteral obstruction (UUO) mice. In addition, IL-33 was constitutively expressed throughout the kidney in peritubular and periglomerular spaces of mice kidneys (Ferhat et al., 2018).

With respect to the IL-33 receptors, ST2L is selectively expressed by several immune cells involved in type 2 immune responses, such as macrophages, invariant natural killer T (iNKT) cells, mast cells, and lymphocytes (Kaur et al., 2015; Lu et al., 2015; Larsen et al., 2018; Kaur et al., 2020). The sST2 is mainly expressed in fibroblasts and epithelial cells (Akimoto and Takenaga, 2019). In the kidney, ST2 is strongly expressed in the glomeruli, renal tubules, and peritubular capillaries in patients with diffuse cutaneous systemic sclerosis (Manetti et al., 2010). Additionally, Yu et al. (2021) also reported ST2 may be localized to renal tubules and interstitial spaces. Another study reported that ST2 is expressed in macrophages, bipotent T and innate lymphoid cells (T/ILC), neutrophils, and monocytes in kidneys of mice after obstructive injury (Chen et al., 2018b). However, the localization of ST2 to other non-immune cells in murine kidneys has not been completely elucidated, and it requires further investigation.

### The Role of IL-33/ST2 Pathway in Renal Fibrosis

The IL-33/ST2 pathway is widely involved in the fibrosis of various organs, including the airway, lungs, heart, intestine, liver, and skin (Table 1). As shown in Table 1, the role of this pathway in cardiac fibrosis is controversial. Sanada et al. (2007) observed that the IL-33/ST2 pathway limits overall ventricular fibrosis, whereas Ghali et al. (2020) reported IL-33 aggravated the deterioration of cardiac function and cardiac remodeling. In fact, it is associated with activation of myofibroblasts and an increase in the pro-fibrotic markers, such as connective tissue growth factor (CTGF) and TGF-β (Ghali et al., 2020). Overall, in majority of fibrotic diseases, this pathway mainly promotes inflammatory responses and facilitates organ fibrosis, ultimately leading to organ dysfunction.

**Table 1:** The role of IL-33/ST2 in fibrosis of various organs.

Fibrotic organ	Disease	Findings	References
Respiratory fibrosis	Asthma	Knockdown of ST2 reduces IL-33-induced collagen I, III, and fibronectin expression, thereby reducing ECMs deposition	An et al., 2018
	Idiopathic pulmonary fibrosis	The levels of ST2, MyD88, and TRAF6 proteins in bleomycin-induced pulmonary fibrosis tissues were elevated.	Xu et al., 2016
	Pulmonary inflammation	IL-33 promotes initiation and progression of pulmonary fibrosis by M2-like polarization of macrophages through ST2 signaling.	Fanny et al., 2018
Cardiac fibrosis	Cardiomyocyte hypertrophy	IL-33 is mechanically induced in cardiac fibroblasts and antagonizes hypertrophic stimuli. IL-33 blocks NF-κB activation by angiotensin II and phenylephrine.	Sanada et al., 2007
	Myocardial infarction	IL-33 aggravated the deterioration of cardiac function, which is associated with activated myofibroblasts and a increase in pro-fibrotic markers, such as connective tissue growth factor (CTGF), and TGF-β.	Ghali et al., 2020
Intestinal fibrosis	Crohn’s disease	IL-33/ST2 pathway may lead to upregulation of TGF-β and facilitate collagen deposition in fibroblasts.	Imai et al., 2019
Liver fibrosis	Chronic CCL4 dependent hepatic fibrosis	IL-33 is released in response to chronic hepatocellular stress. And extracellular IL-33, leads to accumulation and activation of ILC2 in the liver *via* ST2-dependent signaling. Activated hepatic ILC2 produce IL-13, which in turn triggers activation and trans-differentiation of hepatic stellate cells (HSCs).	Marvie et al., 2010
	Biliary atresia (BA)	IL-33/ST2 pathway is correlated with liver fibrosis progression in BA patients, and mast cells participate in this process.	Liu et al., 2019b
	Hepatic inflammation	IL-33/ST2 pathway may activate HSCs *via* MEK/ERK/p38-MAPK signaling.	Tan et al., 2018
Cutaneous fibrosis	Skin inflammation	IL-33 induced fibrosis in an IL-13–dependent manner. And, IL-33 induced skin fibrosis is dependent on eosinophils.	Rankin et al., 2010

Diabetes (Abd Rachman Isnadi et al., 2018), obstructive nephropathy (Martínez-Klimova et al., 2020), ischemia-reperfusion injury (IRI; Rossi et al., 2021), and lupus nephritis (Davidson, 2016) promote the development of CKD. In fact, there are many publications showing that the IL-33/ST2 pathway exacerbates the structural and functional damage of the kidneys in CKD (Table 2). Specifically, in studies of UUO model that is classical renal fibrosis model (Chevalier et al., 2009; Martínez-Klimova et al., 2019), IL-33 inhibited fibrosis in the early stages (Gatti et al., 2021). However, continuous stimulation resulted in significant upregulation of the IL-33/ST2 pathway (Chen et al., 2016; Li et al., 2019b; Gatti et al., 2021). In a word, the progression of renal disease is associated with the sustained activation of IL-33/ST2. Therefore, the IL-33/ST2 pathway appears to be a significant mechanism underlying renal fibrotic disease.

**Table 2:** Advancements in the study of IL-33/ST2 in chronic kidney disease (CKD).

Disease	Renal IL-33 level	Renal ST2 level	Findings	References
Diabetic nephropathy (DN)	Protein↑ mRNA↑	Protein↑ mRNA↑	IL-33/ST2 pathway aggravates renal functional and structural damage by promoting NF-κB p65 pathway, TGF-β, TNF-α, and IL-1β.	Elsherbiny et al., 2020
	Protein↑		IL-33 level is elevated in DN rats with contrast-induced nephropathy. Inhibition of IL-33 provided functional and histological protection.	Onk et al., 2016
**Non-diabetic nephropathy**				
Chronic obstructive nephropathy	mRNA↑	mRNA↑	Elevated IL-33 is devastating renal injury and limit proliferation of tubular epithelial cells.	Chen et al., 2016
	mRNA↑		Nuclear IL-33 in fibroblasts inhibits the initial pro-fibrotic response, but continued stimulation by UUO and secretion of IL-33 exerts a pro-fibrotic effect *via* activated fibroblasts.	Gatti et al., 2021
	mRNA↑	mRNA↑	IL-33 promotes renal fibrosis through macrophages and increases secretion of IL-13 and TGF-β1.	Li et al., 2019b
Acute kidney injury (AKI)			Endogenous IL-33 contributes to kidney IRI by promoting iNKT cell recruitment and cytokine production.	Ferhat et al., 2018
**Immune nephropathy**				
Chronic allograft injury after kidney transplantation	Protein↑		IL-33 may contribute to the development of kidney interstitial fibrosis *via* the p38 MAPK signaling pathway.	Xu et al., 2018
Lupus nephritis	Protein↑ (serum)		IL-33 blockade may have a therapeutic effect on SLE by inhibiting the production of inflammatory cytokines, such as IL-1β, IL-6, and IL-17.	Li et al., 2014b

### Mechanisms of IL33/ST2 Involvement in Renal Fibrosis

During chronic kidney injury and inflammation, endothelial cells, epithelial cells, and fibroblasts release IL-33 (Tonacci et al., 2019). This IL-33 activates ST2-expressing cells, such as polarized M2 macrophages, innate lymphoid type 2cells (ILC2s), CD4^+^ T cells, and iNKTs, thereby triggering inflammation and tissue repair responses. It has been reported that macrophages play an important role in obstructive kidney injury (Meng et al., 2014). Interestingly, IL-33, along with other cytokines, promotes the transformation of M0 macrophages into M2 macrophages (Liew et al., 2016), and persistence of these M2 macrophages contributes to fibrosis (Mosser and Edwards, 2008; Kim et al., 2015). Moreover, Li et al. (2019b) discovered that during UUO-induced renal fibrosis, IL-33 exacerbates renal injury and promotes renal fibrosis *via* the activity of M2 macrophages and an increased secretion of IL-13 and TGF-β1. Incidentally, ILC2s are activated in damaged kidneys; for instance increased amounts of ILC2s have been reported in the kidneys of UUO mice models (Chen et al., 2018b). Additionally, ILC2s promote lung and liver fibrosis, and they can secrete Th2-specific cytokines, such as IL-4 and IL-13 (Liu et al., 2019a), which, in turn, exhibit significant pro-fibrotic activity (Fertin et al., 1991; Wynn, 2003). However, one study reported that IL-33 for a short period of time may attenuate the IRI-induced renal damage *via* ILC2 activity (Riedel et al., 2017; Cao et al., 2018). This may have occurred due to the short duration and low dose of treatment with recombinant IL-33 (rIL-33). Akcay et al. (2011) demonstrated that a sustained high level of rIL-33 can exacerbate renal damage, but they did not specify the role of ILC2s in this process. Therefore, the role of ILC2s, stimulated by sustained high levels of rIL-33, in cases of renal fibrosis requires further investigation. Furthermore, IL-33 can also drive the activation of iNKTs and the infiltration of neutrophils into inflamed tissues (Bourgeois et al., 2011; Thierry et al., 2014; Ferhat et al., 2018). In fact, Ferhat et al. observed that IL-33 promotes IFN-γ/IL-17A production by iNKT cells in IRI, further amplifying the renal tissue injury (Ferhat et al., 2018). The CD4^+^ T cells are also vital for the progression of renal fibrosis (Liu et al., 2012). A previous study has reported that the infiltration of renal CD4^+^ T cells was reduced in acute kidney injury (AKI) mice model injected with sST2. Moreover, in wild-type mice, rIL-33 exacerbated AKI and raised levels of C-X-C motif ligand 1 (CXCL1), but similar results were not observed in CD4 deficient mice (Akcay et al., 2011). These results suggest that the deleterious effects of IL-33 are probably mediated *via* CD4^+^T cells.

In addition to effects of the IL-33/ST2 pathway on the abovementioned immune cells, a few studies also reported its effects on non-immune cells. IL-33 is involved in post-transplant interstitial fibrosis by activating the p38 MAPK signaling pathway and promoting epithelial-mesenchymal transition (EMT) in HK-2 cells (Xu et al., 2018). In fact, Chen et al. found elevated expression of IL-33 in mesenchymal myofibroblasts and perivascular endothelial cells of the renal tubules. They also confirmed that IL-33 deletion *via* gene knockdown reduced UUO-induced renal fibrosis (Chen et al., 2016). These observations suggest that the upregulation of the IL-33/ST2 pathway in case of obstructed kidney disease may promote tubular cell injury and interstitial fibrosis. Another study reported that upregulation of the IL-33/ST2 signaling pathway in case of systemic lupus erythematosus (SLE) promoted renal tubular cell injury and fibrosis primarily through renal EMT (Chen et al., 2017). Moreover, Liang et al. (2017) found that IL-33 worsened renal fibrosis after IRI. On the contrary, sST2 significantly inhibited collagen deposition in the kidneys of IRI-stressed mice by inhibiting IL-33. It is possible that IL-33 recruits bone marrow-derived fibroblasts and inflammatory cells into the kidney, thereby producing proinflammatory factors and profibrotic molecules (Liang et al., 2017). Similarly, Zhu et al. found that IL-33 induced the phenotypic transformation of bone marrow-derived monocytes into fibroblasts in a dose-dependent manner, thereby leading to a marked increase in the expression of α-SMA and fibronectin (Zhu et al., 2019).

**Figure 2:**
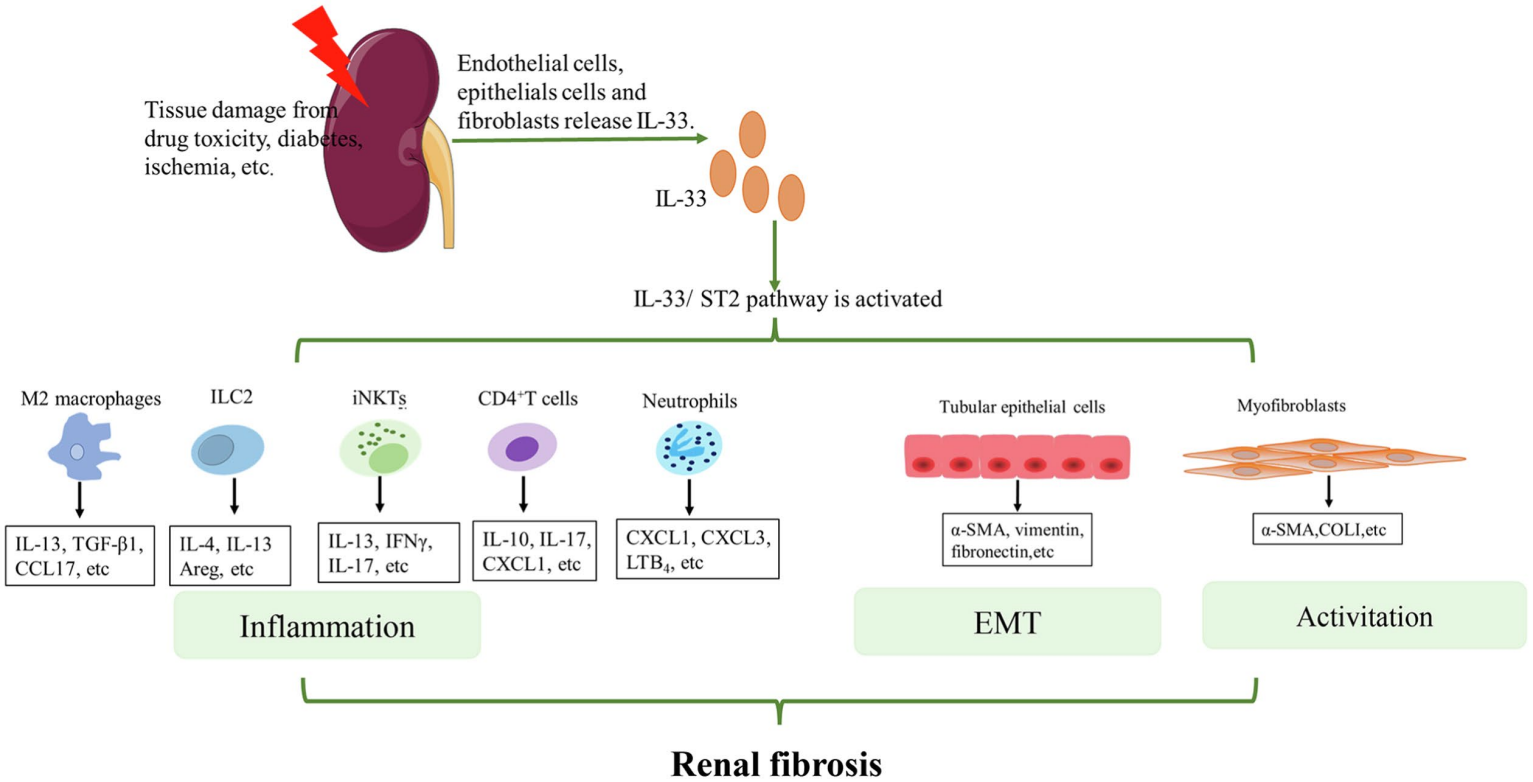
Mechanisms of IL-33/ST2 involvement in renal fibrosis. When the kidney is injured, endothelial cells, epithelial cells, and fibroblasts release IL-33. IL-33 binds to ST2L on the cell membrane and activates downstream pathways. For immune cells, such as M2 macrophages, ILC2s, iNKTs, CD4^+^T cells, and neutrophils, IL-33 activates these cells and promotes the secretion of some cytokines that further aggravate inflammation. For other non-immune cells in the kidney, IL-33 can lead to EMT of renal tubular epithelial cells and activation of myofibroblasts. Eventually, COL1, α-SMA etc. are produced. Continued inflammation, EMT, and activation of myofibroblasts finally lead to renal fibrosis.

Generally, the mechanisms of the IL-33/ST2 pathway in the progression of renal fibrosis can be summarized in two aspects (Figure 2). On the one hand, this pathway promotes the secretion of pro-inflammatory and pro-fibrotic factors, such as IL-4, IL-13, and TGF-β by some immune cells. These cytokines promote inflammation. Notably, TGF-β is an important central mediator of renal fibrosis (Meng et al., 2015). Some previous studies found that IL-33 expression was positively correlated with TGF-β (Li et al., 2014a; Kotsiou et al., 2018; Elsherbiny et al., 2020). This may be related to macrophages (Li et al., 2019b) or ILC2 (Nagashima and Iyoda, 2021). The specific link and mechanism between IL-33/ST2 and TFG-β need to be investigated further. On the other hand, the IL-33/ST2 pathway can promote fibrogenesis by triggering EMT of renal tubular epithelial cells and activating myeloid fibroblasts. Ultimately, these changes promote the secretion of collagen and fibronectin to facilitate fibrogenesis. However, the effect of this pathway on other non-immune cells of the kidney, such as collecting duct cells and podocytes, is unknown and requires further research.

## The Potential Value of the IL-33/ST2 Pathway for Renal Fibrosis

### The IL-33/ST2 Pathway: A Promising Biological Marker of Kidney Disease

Patients with kidney disease lack clinical symptoms and have low sensitivity to serum creatinine (Cr) tests in the early stages. Moreover, a decreased glomerular filtration rate is usually detected in the moderate or severe stages of CKD; this means that patients miss the optimal period for treatment. Early detection and correction of modifiable risk factors can decrease the rate of decline in kidney function (Ruggenenti et al., 1999; McClellan and Flanders, 2003; de Zeeuw et al., 2006; Ho et al., 2013). Therefore, researchers aim to identify novel biological markers in patients with kidney disease that will allow early detection and treatment of the condition.

Currently, IL-33 and ST2 are used as biological markers mainly in patients with heart disease. In fact, sST2 has been shown to independently predict heart failure (HF), major cardiovascular events, and mortality of the patients (Seo et al., 2018; Alam et al., 2019; Homsak and Gruson, 2020). Incidentally, levels of cardiac ST2 mRNA and serum ST2 protein are known to increase in patients with myocardial infarction (Weinberg et al., 2002). Additionally, IL-33 has been associated with vascular disease dysfunction (Gungor et al., 2017). It has been reported that IL-33 effectively activates the type 2 cytokine milieu in the damaged heart and is associated with the deterioration of cardiac function as well as cardiac remodeling (Ghali et al., 2020). Furthermore, serum IL-33 and sST2 levels were elevated in patients with chronic HF (Zhang et al., 2012), and they continue to increase with the degradation of cardiac function (Xiang et al., 2021). What is more, the expression of IL-33 and ST2 in myocardial tissue from patients with end-stage HF was reported to be elevated. In addition, their levels were significantly correlated with the degree of cardiac fibrosis and the expression level of TGFβ1 (Tseng et al., 2018). It is evident that the IL-33/ST2 pathway plays a regulatory role in the progression of cardiac fibrosis, thereby making it a useful tool for the prognosis and diagnosis of heart failure.

It is well-known that the heart and kidneys are closely linked organs under physiological and pathophysiological conditions (Palazzuoli et al., 2015). Acute or chronic heart disease can directly lead to the deterioration of acute or chronic renal function and vice versa. This phenomenon referred to as the cardiorenal syndrome. Incidentally, the coexistence of cardiac and renal dysfunction can significantly increase the mortality and morbidity of the patients, as well as cost of patient care (Ronco et al., 2010; Cruz et al., 2011; Palazzuoli et al., 2015). HF is the main cardiovascular complication and the leading cause of mortality in patients with renal disease, especially the ones with ESRD (Bansal et al., 2017). It is clear that kidney disease and heart disease are closely related. Therefore, given that serum IL-33 and sST2 levels are known to reflect heart function, some researchers have investigated whether they might also reflect kidney function. One study reported that circulating levels of sST2 are independently and negatively associated with a poor diuretic response in patients with acute HF and renal dysfunction (Liu et al., 2019b). Another study revealed that serum IL-33 is a sensitive marker of renal function in patients with gout (Duan et al., 2016). Gungor et al. (2017) reported that IL-33 and ST2 levels increase with the gradual progression of the CKD stages. In fact, in a cohort study of critically ill patients, the ones with elevated sST2 expression had the worst prognosis (Dieplinger et al., 2016). However, some studies indicate that sST2 is a better indicator of risk for end-stage dialysis than IL-33 in patients with kidney disease (Bao et al., 2012; de Boer et al., 2015; Tuegel et al., 2018; Lukic et al., 2020; Yan et al., 2020). For instance, Bao et al. (2012) did not observe any difference in the serum IL-33 concentrations of patients with CKD and that of healthy individuals. However, sST2 serum level was elevated in patients. Furthermore, a significant correlation between the sST2 levels and the disease severity was found by correlation analysis (Bao et al., 2012). Similarly, Lukic et al. (2020) reported that sST2, but not IL-33, is associated with ESRD parameters, and the serum sST2 concentration is positively correlated with that of serum urea and creatinine. Hence, it is unclear whether IL-33 can be used as a reference indicator in patients with kidney disease. However, sST2 appears to be a promising biological marker for kidney diseases, and deserves further study and wide application in clinical practice.

### The IL-33/ST2 Pathway as a New Therapeutic Target for CKD

Patients with ESRD require dialysis or kidney transplantation (Hewitson, 2009). However, this treatment modality is limited in the developing and underdeveloped countries due to the high costs (Nogueira et al., 2017). At present, glucocorticoids are the mainstay of treatment for kidney disease. However, their widespread clinical use is largely limited because of their association with a variety of serious adverse effects, such as infections, hypertension, and osteoporosis (Ponticelli and Locatelli, 2018; Vandewalle et al., 2018). Therefore, it is necessary to identify new therapeutic targets and medicines to prevent disease progression and ultimately death.

From the above discussion, it is evident that the IL-33/ST2 pathway plays a key role in renal fibrosis. In particular, *in vivo* studies in IL-33-deficient transgenic mice further supported the importance of the IL-33/ST2 pathway in the progression of renal fibrosis. For instance, the damage to the proximal tubules was reduced in the kidneys of the IL33−/− UUO model mice (Chen et al., 2016). Similarly, compared with wild-type mice, IRI mice lacking IL-33 showed reduced levels of tubular cell injury (Ferhat et al., 2018). Moreover, the SLE mice treated with anti-IL-33Ab, which has a protective effect on SLE, showed lower serum and renal levels of IL-1β, IL-6, and IL-17 than that in the untreated mice (Li et al., 2014b). Therefore, the IL-33/ST2 pathway may form a new therapeutic target for CKD.

In this regard, IL-33 blockers may be a novel treatment modality for CKD patients; for instance, the monoclonal antibody blockade of IL-33 is a novel immunotherapy agent. Incidentally, AstraZeneca is conducting the trial of a monoclonal antibody called MEDI3506 (anti-IL-33) in patients with diabetic nephropathy. However, this trial has not yet been completed (Jiang et al., 2021). It is known that the main side effect of monoclonal antibody administration is the risk of an adverse immune response (Hansel et al., 2010; Liu and Li, 2014). Therefore, this method requires extensive research. In addition, IL-2 and IL-33, especially as a hybrid cytokine (IL233-bearing IL-2 and IL-33 activities in one molecule), potentiate Tregs and ILC2s to prevent renal injury (Sabapathy et al., 2019). However, this method may be suitable only on a short-term basis as IL-33 becomes a deleterious factor with prolonged stimulation (Akcay et al., 2011).

Traditional Chinese Medicine (TCM) and other natural plant compounds offer certain therapeutic advantages over standard medicine, such as potentially lower prices and ease of access. Therefore, these form a good choice for the development of new medicines. Incidentally, the use of TCM has been extensively studied in cases of renal fibrosis (Table 3). However, there is limited information about TCM targeting the IL-33/ST2 pathway in the treatment of kidney diseases. Interestingly, Calycosin, an isoflavone, is the predominant component of Radix Astragali, which has been widely administered to ameliorate the symptoms of diabetes and diabetic nephropathy (Zhang et al., 2019). Calycosin treatment significantly reduced mRNA expression and protein levels of IL-33 and ST2 when compared to diabetic rats. Thereby, it affected the downstream pathway, p65 NF-κB. Finally, it delayed progression of renal fibrotic events (Elsherbiny et al., 2020). In addition, astragaloside IV (AS-IV; Bao et al., 2016), Cnidium Cnidii (Yang et al., 2020), and salidroside (Cai et al., 2020) have also been shown to reduce IL-33 to improve the disease condition. However, these studies were conducted in non-renal diseases, such as allergic inflammation. AS-IV, Cnidium cnidii, and salidroside have therapeutic effects on renal fibrosis (see Table 3). Thus, the role of these TCMs on IL-33/ST2 signaling in the kidney remains to be investigated.

**Table 3:** Mechanistic studies of Traditional Chinese Medicine (TCM) in the treatment of renal fibrosis.

TCM	Animal model	Drug dose	Mechanism	References
Huangqi-Danshen Decoctio	Adenine-induced kidney disease	4.7 g/kg/day	Kinetic-related protein 1expression↓ Mitogenic protein 2 expression↑ Mitochondrial dynamics restoration	Liu et al., 2019c
Yiqihuoxue Formula	Adenine-induced kidney diseas	12 g/kg/day	LC3-II and Beclin-1 expression levels↑ P62 expression levels↓	Xia et al., 2019
ErHuang Formula	Diabetic nephropathy	4,2, and 1 g/kg/day	CXCL6/JAK/Stat3 pathway↓	Shen et al., 2019
Tongxinluo	Diabetic nephropathy	0.75 g/kg/day	TGf-β/Smad3 pathway↓	Wu et al., 2017
Astragaloside IV	Diabetic kidney disease	40 mg/kg/day	SIRT1, autophagy↑	Wang et al., 2019b
Salidroside	Unilateral ureteric obstruction	40 and 80 mg/kg	TLR4/NF-κB pathway↓ MAPK pathway↓	Li et al., 2019a
Isoliquiritigenin	Unilateral ureteric obstruction	7.5 and 30 mg/kg	Mincle/Syk/NF-κB pathway↓	Liao et al., 2020
Osthole	Unilateral ureteric obstruction	40 and 80 mg/kg/day	TGF-β/Smad pathway↓ NF-κB pathway↓	Zhang et al., 2018
Huangkui capsule	Unilateral ureteric obstruction	0.15, 0.5, and 1.5 g/kg	TRPC6 channel activity↓	Gu et al., 2020b
Shenkang injection	Unilateral ureteric obstruction	5 g/kg/d, 1 g/kg/d	PDGFR pathways↓ VEGFR pathways↓	Liu et al., 2019e
Abelmoschus manihot(L.) Medik	5/6 nephrectomy	0.15, 0.5, and 1.5 g/kg	PI3K-AKT pathway↓ ERK1/2 pathway↓	Gu et al., 2020a
ShenShuai II Recipe	5/6 nephrectomy	10 ml/kg/day	Sirt1/Smad3 deacetylation pathway↑ NLRP3/ASC/Caspase-1/IL-1β↓	Wang et al., 2019a

Other natural plant compounds can also attenuate IL-33 expression. Chrysin (CR) is one of the flavonoids that are commonly used as a traditional medicine and found in many plant extracts. CR has an ameliorative effect on CKD (Ali et al., 2015). Moreover, CR reduced PbAc-induced renal inflammation and significantly reduced renal IL-33 levels (Kucukler et al., 2021). Zingerone (ZO), a component of dry ginger root, has several pharmacological activities owing to its antioxidant, anti-inflammatory and anti-apoptotic properties. Kandemir et al. (2018) demonstrated that ZO remarkably reduced the levels of IL-33 in VCM-induced nephrotoxicity.

In conclusion, blocking the IL-33/ST2 pathway may be a novel therapeutic strategy for treating renal diseases in the future. Calycosin, CR, and ZO can attenuate IL-33 to protect renal function, thereby making them potential candidates for developing new medicines. Other effective natural compounds targeting IL-33/ST2 still need to be discovered for the treatment of kidney diseases.

## Conclusion

Kidney disease is a major public health problem worldwide. Although kidney transplantation and dialysis treatments are available, there is a significant lack of effective treatments. Therefore, in recent years, researchers have focused on the identification of new treatment targets for patients suffering from kidney diseases. Gradually, the role of the IL-33/ST2 pathway in fibrotic diseases has been established. Although its effects have been extensively studied in cardiac, pulmonary, and liver fibrosis (Willems et al., 2012; Sun et al., 2017; Drake and Prakash, 2020), research related to renal fibrosis still in the nascent stages. In this review, we have focused on discussing the role of the IL-33/ST2 pathway in the development and progression of renal fibrosis as well as the underlying mechanisms of this process. Incidentally, IL-33 activates the immune cells and promotes the secretion of certain cytokines, further aggravating inflammation. However, in other non-immune cells of the kidney, IL-33 can lead to EMT of renal tubular epithelial cells and activation of myofibroblasts. Additionally, many clinical studies have revealed that the IL-33/ST2 pathway may be an effective biomarker of kidney disease, and this is important for the early diagnosis of CKD and assessment of patient prognosis. However, further detailed and comprehensive studies are required for addressing the existing gaps in knowledge. Meanwhile, the IL-33/ST2 pathway appears to be a promising therapeutic target for renal fibrosis. TGF-β, a strong pro-fibrotic factor, and its relevant pathway have been extensively studied in cases of renal fibrosis (Loboda et al., 2016). However, IL-33 is a newly discovered pro-fibrotic factor. Hence, information related to the IL-33/ST2 pathway is still limited, and medicine targeting the IL-33/ST2 signaling is yet to be developed. We hope this review will contribute to the comprehensive studies of the IL-33/ST2 pathway in renal fibrosis and help in the development of novel therapeutic agents.

## Author Contributions

X-YT designed the ideas and wrote the first version of the manuscript. H-YJ contributed to resources. X-YT and H-YJ have contributed equally to this work. Y-RM reviewed the draft of the manuscript. All authors contributed to the article and approved the submitted version.

## Funding

The study was sustained by National Natural Science Foundation of China (81973732).

## Conflict of Interest

The authors declare that the research was conducted in the absence of any commercial or financial relationships that could be construed as a potential conflict of interest.

## Publisher’s Note

All claims expressed in this article are solely those of the authors and do not necessarily represent those of their affiliated organizations, or those of the publisher, the editors and the reviewers. Any product that may be evaluated in this article, or claim that may be made by its manufacturer, is not guaranteed or endorsed by the publisher.

## Figures and Tables

**Table:** 

AS-IV	Astragaloside IV
CKD	Chronic kidney disease
CR	Chrysin
ECM	Extracellular matrix
EMT	Epithelial-mesenchymal transition
ESRD	End-stage renal disease
IL-1	Interleukin 1
IL-33	Interleukin 33
IL-1RACP	IL-1 receptor accessory protein
ILC2	Lymphoid type 2 cells
iNKT	Invariant natural killer T
IRI	Ischemia-reperfusion injury
MyD88	Myeloid differentiation primary response 88
NF-κB	Nuclear factor kappa light chain enhancer of activated B cells
rIL-33	Recombinant IL-33
SLE	Systemic lupus erythromatous
sST2	Soluble ST2
ST2	Suppression of tumorigenicity 2
TCM	Traditional Chinese medicine
TGF-β	Transforming growth factor-beta
Th2	T-helper 2
TRAF6	Tumor necrosis factor receptor (TNFR)-associated factor 6
UUO	Unilateral ureteral obstruction
ZO	Zingerone
